# Stereocontrolled Synthesis and Functionalization of Cyclobutanes and Cyclobutanones

**DOI:** 10.3390/molecules181215541

**Published:** 2013-12-13

**Authors:** Francesco Secci, Angelo Frongia, Pier Paolo Piras

**Affiliations:** Dipartimento di Scienze Chimiche e Geologiche, Università degli Studi di Cagliari, complesso universitario di Monserrato, S.S. 554, bivio per Sestu, Monserrato 09042, CA, Italy

**Keywords:** cyclobutanone, cyclobutane, stereocontrol, ring enlargement, alkylation, organocatalysis, cycloaddition, biocatalysis, stereochemistry, oxidation

## Abstract

In the last decade a certain number of new cyclobutane and cyclobutanone synthesis and functionalization protocols have been published. Organo- and biocatalyzed eco-friendly approaches to cyclobutane-containing molecules have been developed with interesting results. Also, successful new total synthesis of bioactive compounds and drugs have been recently reported where a four membered ring represented the key intermediate. Therefore, the rising interest in this field represents a great point of discussion for the scientific community, disclosing the synthetic potential of strained four membered ring carbocyclic compounds. Herein we report a critical survey on the literature concerning the enantiocontrolled synthesis and functionalization of cyclobutane derivatives, with particular attention to metal-free, low impact methodologies, published during the period 2000–2013.

## 1. Introduction

**Figure 1:**
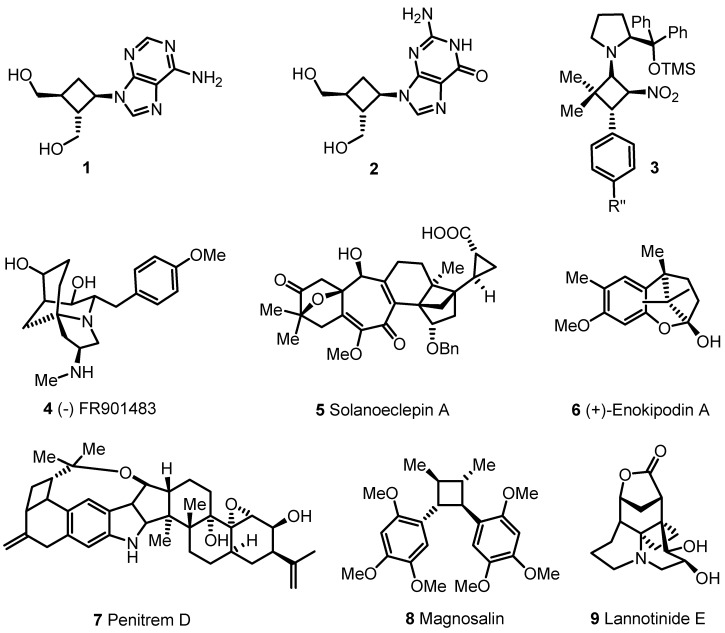
Cyclobutane containing natural products and synthetic intermediates.

Strained carbocyclic molecules have emerged in the past decades as highly useful synthetic tools [1]. In this class of compounds cyclopropane and cyclobutane derivatives certainly represent the most studied and versatile organic molecules [2]. Due to their inherent ring strain, the selective modification of their structures can be strategically used in organic synthesis [3]. Ring enlargement and ring contraction can be obtained regio- and stereoselectively by using a certain number of reaction conditions [4,5]. Moreover carbocyclic ring opening is possible and it represents an advantageous synthetic route to acyclic compounds [6]. Cyclobutane and cyclobutanone derivatives can be easily prepared by reliable synthetic methods [7] such as [2+2] cycloadditions, [8,9] cyclopropanol- [10,11], cyclopropylphenylthio- [12,13,14,15] and selenium-carbinols ring expansions [16,17,18] or ring enlargement of oxaspiropentanes [19,20,21]. A large number of papers and patents have been published in this area. Cyclobutanones have been employed as key starting materials [22] for a wide number of total syntheses [23,24] (examples are the syntheses of compounds **4**–**8** [25,26,27,28], Figure 1) and as building blocks for the preparation of bioactive molecules and drugs such as the cyclobutane nucleosides **1** and **2** [29,30,31,32]. An example of the versatility of this class of molecules is well represented by the squaric acid derivatives, which are involved in a large number of synthetic applications as demonstrated by Moore and co-workers [33,34,35,36,37,38]. Moreover chiral cyclobutane compounds have been recently isolated independently by Seebach [39] and Blackmond [40], as key intermediates **3**, in the organocatalyzed conjugated addition of nitrostyrenes to different carbonyl compounds. Again, cyclobutane core skeletons are frequently identified in alkaloids [41] and secondary plant metabolites such as compound **9** [42,43].

Among the different transformations of the cyclobutane system, the α-functionalization in most cases [44] involves the use of organometallic-based reactions or metal catalyzed transformations [45,46,47,48]. 

The growing attention to ecofriendly procedures, accompanied by the use and the development of new high-performing chiral organic catalysts has changed certain paradigms about the functionalization and transformation of organic compounds. Moreover, organocatalyzed and biosynthetic procedures have deeply influenced the development of new synthetic approaches. As a consequence of this new sensibility, the synthesis and transformation of strained carbocyclic compounds have been revisited and investigated with the aim of achieving green routes for the preparation of these important synthetic tools. Therefore a, remarkable number of procedures have been recently published, showing a rising interest on the development of stereo- and enantioselective metal free methodologies for the synthesis and functionalization of cyclobutanes [49,50]. 

Based on these considerations, this review will highlight some of the most important and recent achievements in this field. The reader of this review should not expect a complete compendium but rather a selection of papers that report the development of new eco-friendly procedures, mainly organocatalized transformations, highlighting applications in the synthesis of biologically active molecules and natural products where cyclobutanone derivatives appear as key starting materials. In graphical schemes, essential precursors or transition states for the relevant cyclobutane derivatives are placed in parentheses whereas non-isolated intermediates are marked with square brackets. As the synthetic application is emphasized, the reader is referred to the original literature for detailed mechanistic considerations. This review is organized according to the following classes of key steps:

2. Stereoselective [2+2] Cycloaddition Synthesis of Cyclobutane Derivatives
  2.1. Stereoselective Synthesis of Cyclobutane Amino Acids
  2.1. Stereoselective Diels-Alder Reactions using Cyclobutenones
3. Enantioselective Stoichiometric Synthesis of Cyclobutane Derivatives
  3.1. Enantioselective Stoichiometric [2+2] Cycloadditions
  3.2. Chiral Allene based [2+2] Asymmetric Cycloadditions
  3.3. Organocatalyzed Enantioselective [2+2] Cycloadditions
  3.4. Iminium-Ion Intermediated [2+2] Cycloaddition of Enals
  3.5. Hydrogen-Bonding Mediated [2+2] Asymmetric Cycloaddition
4. Desymmetrization of cyclobutane and cyclobutanone derivatives
  4.1. Organocatalyzed Bronsted Acids based Desymmetrizations
  4.2. Organocatalyzed aldol based Desymmetrizations Reactions
  4.3. Enaminocatalyzed Reactions of Cyclobutanones with Nitrosobenzene
5. Biocatalytic Resolution of Cyclobutane Derivatives
  5.1. Biocatalytic PPL based Cyclobutanols Resolution by Esterification and Hydrolysis
6. Cyclobutanone α-Functionalization
  6.1. α-Functionalization of Cyclobutanones via SOMO Catalysis
  6.2. Asymmetric SN1 Alkylation of Cyclobutanones
  6.3. Organocatalyzed Aldol Reactions
  6.4. Organocatalyzed Mannich Addition of Cyclobutanones to Glycolates
  6.5. Organocatalyzed Michael Addition of Cyclobutanones to Nitrostyrenes
  6.6. Cyclobutanone α-Heteroatom Functionalization
7. Cyclobutane Ring Enlargement
  7.1. Chiral Non Racemic Cyclobutanes Ring Expansion. Synthesis of Chiral Cyclopentanones
  7.2. Chiral Non Racemic Oxaspirohexanes Ring Enlargement
  7.3. Organocatalyzed Enantioselective Cyclobutanols Ring Enlargement
  7.4. Organocatalyzed Enantioselective Fluorination-Induced Cyclobutanes and Cyclopropanes Ring Expansions
8. Enantioselective Bayer-Villiger Oxidation
  8.1. Enantio- and Diastereoselective Bayer-Villiger Oxidation of Chiral Cyclobutanones
  8.2. Organocatalyzed Enantioselective Cyclobutanone Bayer-Villiger Oxidation
  8.3. Biocatalytic Enantioselective Cyclobutanone Bayer-Villiger

## 2. Stereoselective [2+2] Cycloaddition Synthesis of Cyclobutane Derivatives

### 2.1. Stereoselective Synthesis of Cyclobutane Amino Acids

**Scheme 1:**
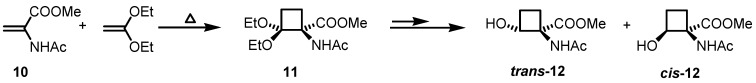
Stereoselective synthesis of (*cis*/*trans*)-2-hydroxycyclobutane amino acids.

Thermal [2+2] stereoselective cycloaddition, involving 2-acylaminoacrylates **10**, as electron-poor acceptor alkenes has been performed by Peregrina and co-workers [51]. This reaction involves a Michael-Dieckmann-type process that allows access to the substituted cyclobutane skeleton **11**. Finally, deacylation and hydrolysis reactions were performed to isolate the 2-hydroxycyclobutane-(*R*)-amino acid serine analogues (c4Ser) **12** as reported in Scheme 1. 

### 2.2. Stereoselective Diels-Alder Reactions using Cyclobutenones

**Scheme 2:**
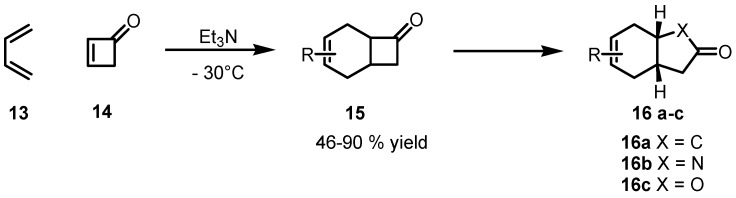
Cyclobutenone as a highly reactive dienophile in Diels-Alder reactions.

Cyclobutenone **14** was employed as dienopile for the first time by Danishesfsky to promote a Diels-Alder cycloaddition reaction with functionalized dienes **13** (Scheme 2). This reaction provides diverse and complex cycloadducts **15** in good yields. Cycloadducts bearing a strained cyclobutanone moiety were able to undergo regioselective ring expansions to produce the corresponding cyclopentanones, lactone, and lactams **16a**–**c**, through a straightforward synthetic approach [52].

## 3. Enantioselective Stoichiometric Synthesis of Cyclobutane Derivatives

### 3.1. Enantioselective Stoichiometric [2+2] Cycloadditions

Photochemical, Diels-Alder reactions and [2+2] ketene cycloadditions represent the most common and efficient routes to cyclobutane or cyclobutanone derivatives. However a big effort has been made in the last years to combine together the possibility to synthetize chiral substituted cyclobutane compounds with high stereo- and enantioselectivity by using chiral auxiliaries or chiral catalysts increasing the atom economy of the synthetic procesess. 

**Scheme 3:**
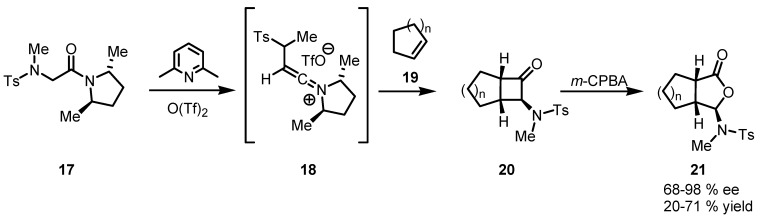
[2+2] Cycloaddition of chiral keteniminium salts in the synthesis of cyclobutanones.

In 2002 Ghosez and co-workers developed an excellent two-step sequence for the asymmetric vicinal acylation of olefins by a [2+2+1] strategy [53]. The key reaction of this methodology is a [2+2] cycloaddition of an olefin **19** to a chiral keteniminium salt **18** derived from N-tosylsarcosinamide **17** yielding stereoselectivelly only *cis*-α-aminocyclobutanones **20** with good enantioselectivity (68%–98% *ee*) through the intermediate **18**. In the original paper, this reaction is followed by a *m*-CPBA regioselective Baeyer-Villiger oxidation providing the lactol derivative **21** in good yields. However, the oxidation step occurs without any detrimental effect on the stereochemistry and enantiopurity of cyclobutanones (Scheme 3).

**Scheme 4:**
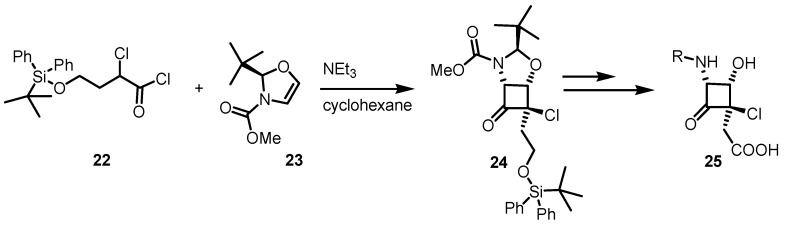
Synthesis of enantiopure α-chlorocyclobutanones.

**Scheme 5:**
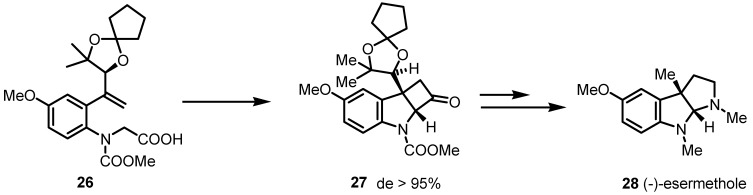
Enantioselective access to pyrrolidinoindoline alkaloids.

A similar approach, was utilized by the same group for the development of another [2+2] ketene mediated cycloaddition reaction between the α-chloroacyl chloride **22** and a chiral oxazolidine **23** affording α-chloro-α'-aminocyclobutanones **24**. The bicyclic cyclobutane derivative **24** was subsequently converted in the enantiopure cyclobutane aminoacid **25** after few synthetic steps [54] as reported in Scheme 4.

(−)-Esermthole (**28** [55]) has been synthetized in 2013 by Shishido and co-workers, through a key [2+2] cycloaddition reaction that simultaneously generated the tricyclic unit **27** in one single synthetic step [56]. The stereochemistry of the fused cyclobutanone **27**, has been controlled by the introduction of a dioxolane chiral auxiliary in the starting material **26**, thus obtaining the chiral scaffold in >95% de. Further functionalization of the cyclobutanone moiety allowed to afford the fused bispyrrolidine natural product (−)-**28** with high enantio- and diastereoselectivity (Scheme 5). 

**Scheme 6:**
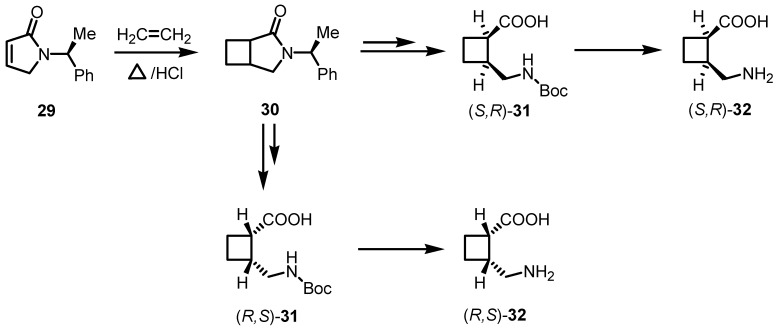
Stereoselective synthesis of *cis*- and *trans*-γ-cyclobutane amino acids.

Aitken and co-workers reported the [2+2] cycloaddition of ethylene with chiral unsaturated γ-lactam **29**. The so obtained cyclobutane derivative **30** was transformed in few synthetic steps in the corresponding Boc-2-aminomethylcyclobutanecarboxylic acids **31** [57]. This synthetic protocol has been modified in a second time by the same group with the aim to improve its efficiency [58]. The synthetic pathway to access to racemic *cis*-cyclobutane γ-amino acid core **30**, reported in Scheme 6, was simplified and the yields were improved [58] Racemic 2-aminocyclobutanecarboxylic acids **32** were diastereoisomerically separated, giving the advantage to afford both of the enantiomers through a non-destructive cleavage of the chiral auxiliary either by hydrolysis or by amonolysis, thus providing an efficacious access to *N*-protected derivatives (*R*,*S*)-**31** and (*S*,*R*)-**31** and their corresponding γ-aminoacids **32**.

### 3.2. Chiral Allene-Based [2+2] Asymmetric Cycloadditions

**Scheme 7:**
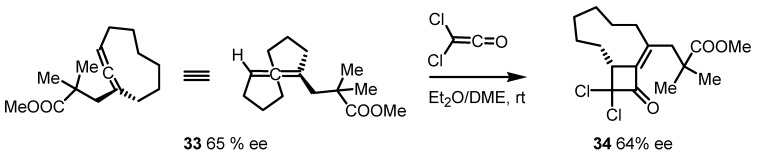
Use of a chiral allene in the synthesis of substituted cyclobutenones.

Endocyclic allene **33**, has been used by Ogasawara and co-workers in the synthesis of chiral cyclobutenone **34** through a [2+2] regio- and stereoselective cycloaddition with dichloroketene. The reaction smoothly proceeds with good stereospecificity affording the scalemic cycloadduct **34** with complete transfer of the optical purity (Scheme 7) [59].

### 3.3. Organocatalyzed Enantioselective [2+2] Cycloadditions

**Scheme 8:**
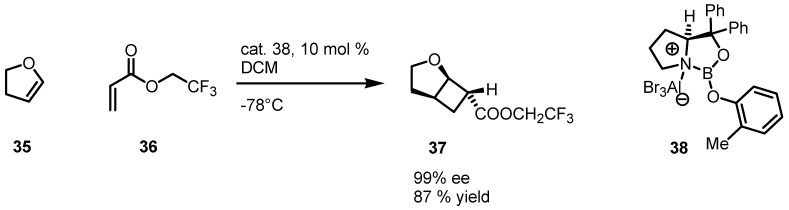
[2+2] enantioselective vinylogous cycloaddition of esters with dihydrofuran.

Corey and co-workers reported in 2007 a straightforward organocatalyzed [2+2] enantioselective vinylogous cycloaddition of esters **36** with dihydrofuran **35** using oxazaborolidine-aluminum bromide complex **38**, conveniently generated *in situ* by the addition of a commercially available solution of aluminum bromide in CH_2_Br_2_ to a cold −20 °C CH_2_Cl_2_ solution of oxazaborolidine (Scheme 8). This procedure afforded the *exo*-[2+2]-cycloadduct **32** in 87% yield and with 99% *ee* [60]. 

### 3.4. Iminium-Ion Intermediated [2+2] Cycloaddition of Enals

**Scheme 9:**
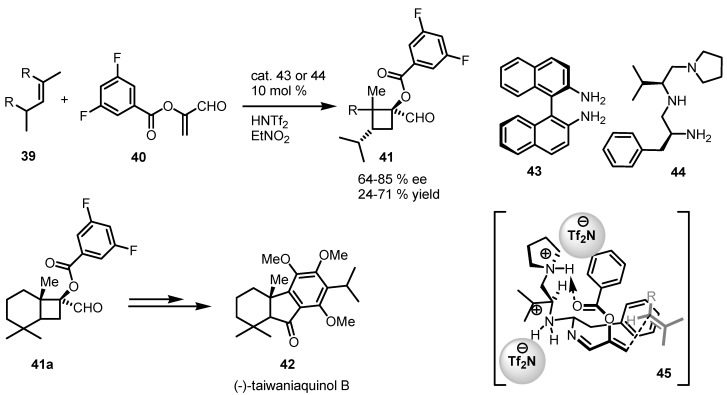
Enantioselective [2+2] cycloaddition of unactivated alkenes with α-acyloxyacroleins.

An enantioselective organocatalytic vinylogous formal [2+2] cycloaddition has been successfully developed based on a tandem iminium–enamine activation of enals [61]. Reactions carried out in the presence of catalysts **43** or **44** and HNTf_2_ gave the enantioselective [2+2] cycloaddition reaction of alkenes **39** and functionalized benzoyloxyacrolein **40** yielding optically active 1-acyloxycyclobutanecarbaldehydes **41**, presumibly through the one of the transition states TS **45**. In the same paper, cyclobutane aldehyde **41a** was also used as chiral intermediate for the synthesis of (−)-taiwaniaquinol B (**42**, [62]) as reported in Scheme 9. 

A [2+2] organocatalytic cycloaddition protocol, has been recently reported by Jørgensen and co-workers [63]. This procedure was efficiently used for the construction of nitrocyclobutanes **48** with four contiguous stereocenters achieving complete diastereo- and enantiomeric control.

This new concept is based on a simultaneous dual activation of α,β-unsaturated aldehydes **46** and nitroolefins **47** via amino- and hydrogen-bonding catalysis. For this purpose, new bifunctional squaramide-based aminocatalyst **49** was synthesized with the idea to enable such an activation strategy. 

**Scheme 10:**
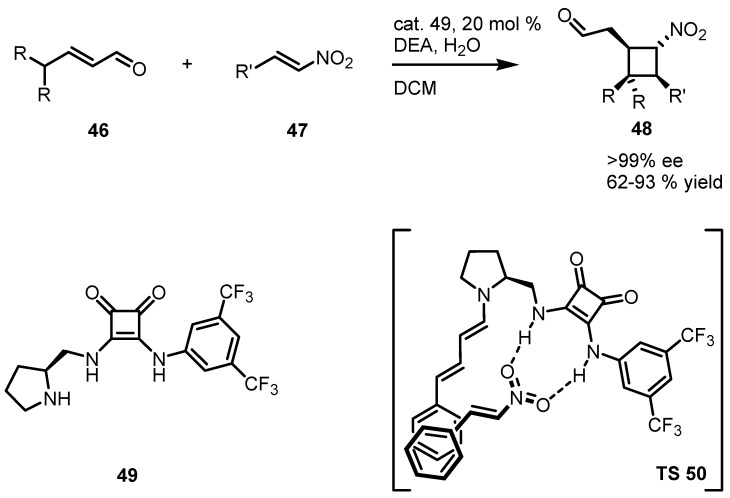
Asymmetric formal [2+2] cycloadditions via bifunctional dienamine catalysis.

The authors reported also an exhaustive computational study that rationalizes the stereochemical outcome of this methodology through the formation of the TS **50** reported in Scheme 10.

**Scheme 11:**
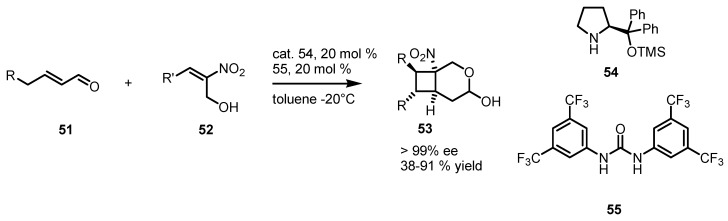
Asymmetric formal [2+2] cycloadditions via enamine catalysis.

Another vinylogous organocatalyzed enantioselective [2+2] cycloaddition based on a similar concept has been reported by Vicario [64], by using diphenyltrimethylsilyloxypyrrolidine **54** and thiourea derivatives co-catalyst **55** as nitrostyrene hydrogen bonding activator (Scheme 11). The procedure, represent a good way to access, from enals **51**, to interesting cyclobutalactols **53** with the creation of four new stereocentres with high *ee* values (85%–94%) and satisfactory yields. 

**Scheme 12:**
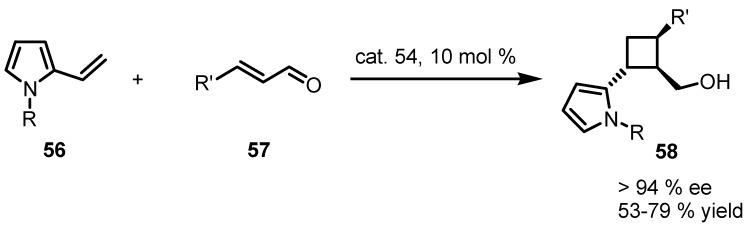
Asymmetric vinylogous formal [2+2] cycloadditions via enamine catalysis.

Catalyst **54** was also employed in an iminium-intermediated cycloaddition of enals **57** and 2-vinyl-pyrroles **56** [65]. The methodology, developed by Xu and co-workers represent an interesting variation of the above mentioned vinylogous [2+2] cycloaddition wherein the nitroalkene was replaced by the use of enals, accessing to chiral pyrrole-cyclobutane derivatives **58** with high stereocontrol of the three new formed stereocentres and accompanied by satisfactory yields as reported in Scheme 12. 

### 3.5. Hydrogen-Bonding Mediated [2+2] Asymmetric Cycloaddition

**Scheme 13:**
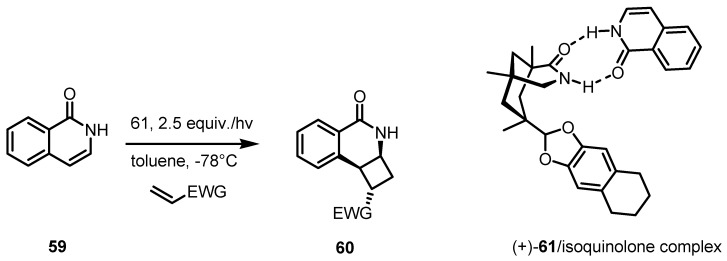
Intermolecular [2+2] cycloaddition of isoquinolone via a chiral H-bonding template.

The first examples of enantioselective intermolecular [2+2] photocycloadditions of isoquinolone **59** with EWG-functionalized alkenes has been reported very recently by Bach and co-workers [66]. Photoreactions were carried out at low temperature via a chiral hydrogen-bonding template **61**. This supramolecular complex, is able to shield one face of the isoquinolone **59**, thus directing the stereochemistry of the [2+2] photocycloadditions. Functionalized tricyclic cyclobutane derivatives **60**, were obtained in excellent yields (86%−98%) and with outstanding regio-, diastereo-, and enantioselectivity as reported in Scheme 13.

## 4. Desymmetrization of Cyclobutane and Cyclobutanone Derivatives

### 4.1. Organocatalyzed Bronsted Acids based Desymmetrizations

In 2010 List and co-workers reported the design and the successful implementation of a new class of chiral binaphthylphosphoric acids-pyridinamides [67] **65** which were used as powerful catalysts in the enantioselective desymmetrization of *meso* anhydrides **62** directing the enantioselective anidride cleavage and the selective esterification of a carboxylic unit yielding compounds **63** in high yields and *ee*.

**Scheme 14:**
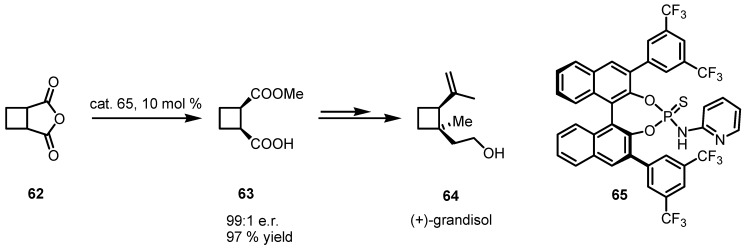
Bifunctional Brønsted acids based desymmetrization of meso cyclobutaneanidrides.

This desymetrization protocol was also used for the synthesis of the boll weevils Anthonomus grandis Boheman pheromone (+)-grandisol (**64**, [68]) as shown in Scheme 14.

### 4.2. Organocatalyzed Aldol based Desymmetrization Reactions

**Scheme 15:**
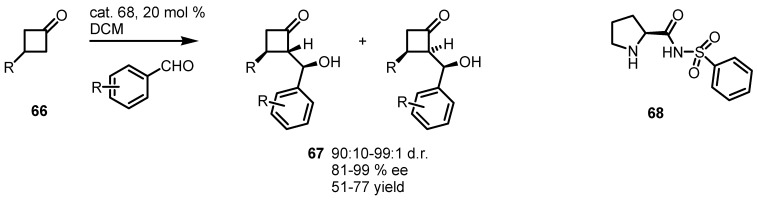
Desymmetrization of 3-substituted cyclobutanones via organocatalyzed aldol reactions.

The enantio- and diastereoselective desymetrization of 3-substituted cyclobutanones **66** has been recently achieved by Frongia and Piras, by using a *N*-phenylsulfonyl-(*S*)-proline **68** catalyzed aldol reaction, affording the corresponding 2,3-functionalized cyclobutanones **67** in good yield and with excellent diastereo- and enantioselectivity [69] as described in Scheme 15. 

### 4.3. Enaminocatalyzed Reactions of Cyclobutanones with Nitrosobenzene

**Scheme 16:**
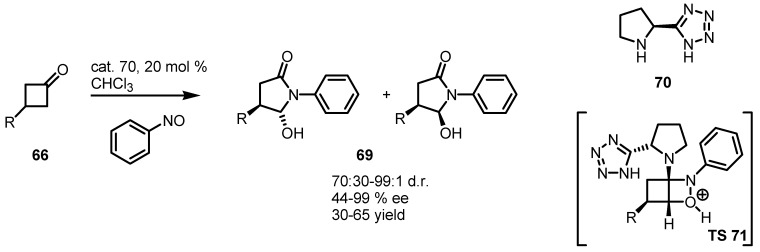
Organocatalyzed synthesis of chiral 4-substituted γ-lactams.

The same group reported another original organocatalyzed enantioselective desymmetrization reaction of 3-substituted cyclobutanones **66** [70]. This desymetrization procedure is based on a tandem *O*-nitrosobenzene alkylation-cyclobutanone ring expansion, mediated by proline derivative catalysts and in particular from the tetrazole derivative **70**, as reported in Scheme 16. In this conditions, cyclobutanones **66** were converted into 4-substituted-5-hydroxy-γ-lactams **69** [71,72] through the TS **71**. The synthetic protocol provides enantiomerically enriched nitrogen containing five-membered ring systems in good yields and *ee* with the generation of two new stereogenic centers. 

## 5. Biocatalytic Resolution of Cyclobutanes

### 5.1. Biocatalytic PPL based Cyclobutanol Resolution by Esterification and Hydrolysis

**Scheme 17:**
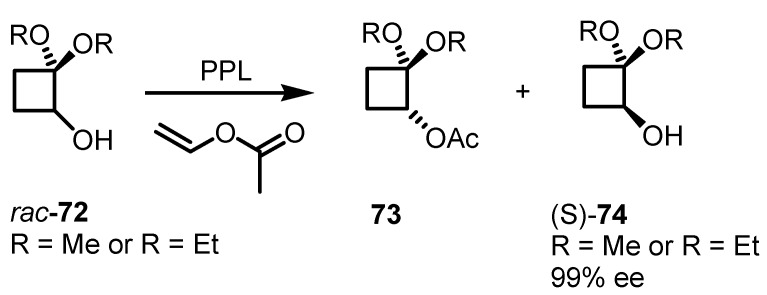
Enzymatic transesterification of cyclobutanols.

Biocatalytic methods for the regio- and enantioselective resolution of cyclobutane derivatives have been recently developed by Fadel and co-workers [73]. Porcine pancreatic lipase PPL is able to discriminate between the two (±)-alcohols *rac*-**72**, affording the optically pure ester **73** and allowing to isolate the cyclobutane alcohol (*S*)-**74** as pure enantiomer as showed in Scheme 17.

**Scheme 18:**
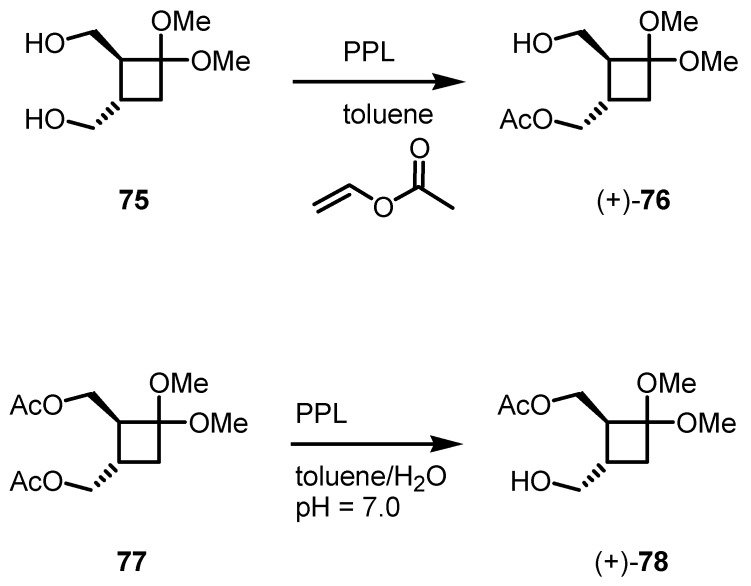
Stereoselective enzymatic esterification and hydrolysis of cyclobutanols.

Another PPL-based cyclobutane resolution has been published by Lee-Ruff [74]. Diole **75** can be efficiently resolved in toluene/vinylacetate to afford the ester **76** as pure compound. Also, cyclobutane diacetate **77** was selectively hydrolyzed from PPL at pH 7.0, in absence of the acetate source, yielding the monoacetate (+)-**78** in 97% yield as reported in Scheme 18. The so obtained derivatives have been afterward used for the synthesis of important key intermediates in the synthesis of chiral cyclobutane nucleosides [30] and aminoacids [31,75,76,77]. 

## 6. Cyclobutanone α-Functionalization

The past few years have witnessed notable breakthroughs in the development of asymmetric intermolecular α-alkylations of carbonyl compounds whereas synthetic applications of cyclobutanones other than ring expansion and fragmentation reactions are rare [2,3]. 

### 6.1. α-Functionalization of Cyclobutanones via SOMO Catalysis

In 2010 the McMillan’s group published the first enantioselective organocatalytic α-allylation of cyclic ketones [78] via singly occupied molecular orbital catalysis (SOMO) [79,80]. Geometrically constrained radical cations, generated from the one-electron oxidation of transiently generated enamines, readily undergo allylic alkylation with a variety of allyl silanes. 

**Scheme 19:**
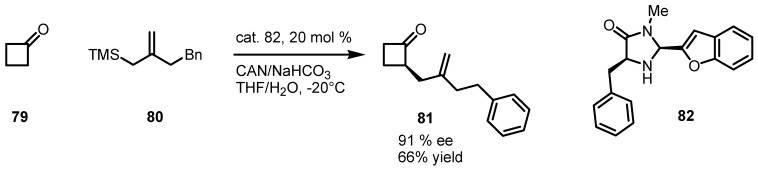
Enantioselective α-alkylation of cyclobutanone via SOMO catalysis.

In this procedure, cyclobutanone **79** was α-functionalized using a new oxidatively stable class of imidazolidinone catalysts, such as compound **82**, and allylsilane **80** in presence of CAN (Scheme 19) to enantioselectivelly afford cyclobutanone **81** in 66% yield. 

### 6.2. Asymmetric S_N_1 Alkylation of Cyclobutanones

**Scheme 20:**
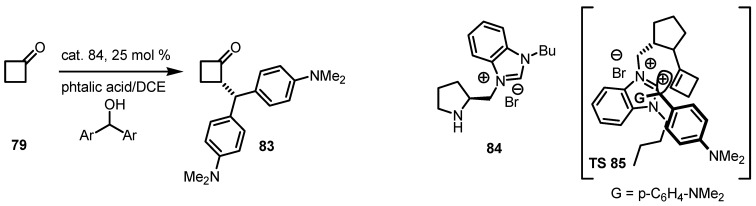
Asymmetric S_N_1 α-alkylation of cyclobutanone catalyzed by chiral ionic liquids.

Cyclobutanone carbocation alkylation, has been recently achieved by Zhang and co-workers [81]. The strategy developed by this group consists in a Brønsted acid *in situ* carbocation generation, using highly polar and ionic liquids and benzoimidazolium derivatives **84** as catalysts. In these experiments, cyclobutanone **79** was reacted with diphenylmethanol, using phtalic acid as additive affording α-functionalized cyclobutanone **83** in good yields and satisfactory enantiomerical excess. FCILs might provide a favorable catalytic sphere for direct α-alkylation of ketones, in which ionic intermediates, such as **86**, are involved through an asymmetric S_N_1 alkylation [82] as reported in Scheme 20. 

### 6.3. Organocatalyzed Aldol Reactions

**Scheme 21:**
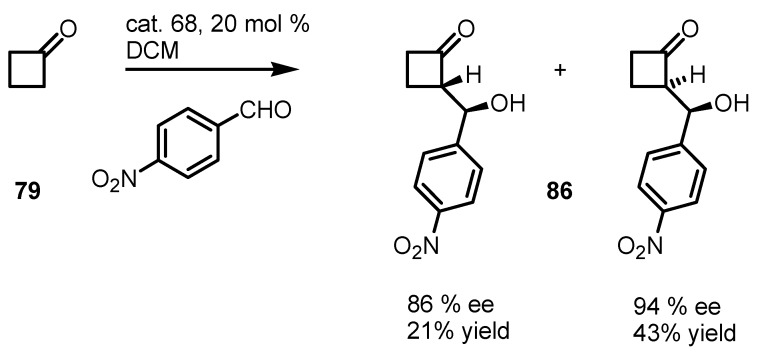
Enantioselective organocatalyzed synthesis of cyclobutanone aldol derivatives.

Cyclobutanone aldol reactions were explored for the first time by Ley and co-workers in 2005 using (*S*)-proline-*N*-phenylsulfonamide organocatalysts **68** and the pyrrolidinetetrazole derivative **70** [83]. These catalysts were developed as valid alternative to (*S*)-proline based catalysis, overcoming, solubility and solvent problems [84,85]. Good results were obtained when catalyst **68** was used in the direct aldol reaction between cyclobutanone **79** and *p*-nitrobenzaldehyde affording the corresponding *syn*/*anti* adducts **86** in high enantiomerical excess and reasonable diastereoselectivity (Scheme 21). 

**Scheme 22:**
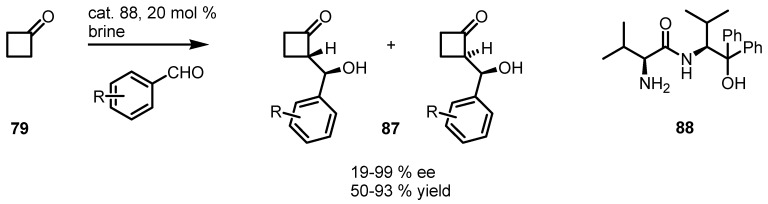
Enantioselective organocatalyzed synthesis of cyclobutanone aldol derivatives.

Similar results were achieved by Ma’s group using catalytic systems made up of primary amine organocatalysts, derived from natural primary amino acids **88**, in combination with 2,4-dinitrophenol (DNP) as additive [86]. Catalyst **88** have proven to be an efficient catalyst in the direct aldol reactions of cyclobutanone **79** with different aromatic aldehydes, in brine without further addition of organic solvents (Scheme 22), affording the corresponding aldol adducts **87** in good yields and high *ee* (d.r. up to 1:99). 

**Scheme 23:**
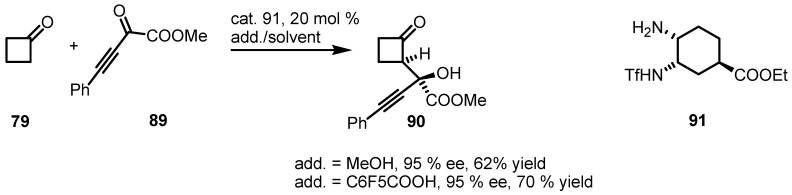
Enantioselective organocatalyzed synthesis of cyclobutanone aldol derivatives.

Interesting results have also been achieved by Maruoka and co-workers [87] in the alkylation of cyclic ketones using primary amine catalysts **91** through a scrupulous screening of additives. Reaction of cyclic ketones including cyclobutanone **79** (Scheme 23) with α-oxoalkinyl esters **89** in the presence of bifunctional primary amine catalyst **91** and achiral acid additives afforded *syn*-aldol adduct **90** with good yields and excellent enantiomeric excess.

**Scheme 24:**
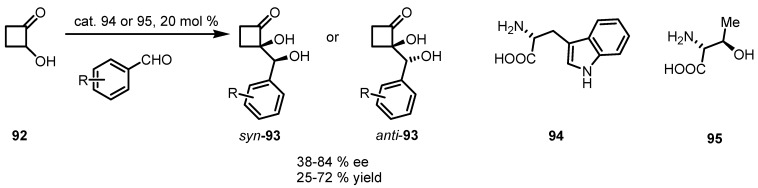
Enantioselective organocatalyzed synthesis of *syn*- and *anti*-cyclobutanone diols.

**Figure 2:**
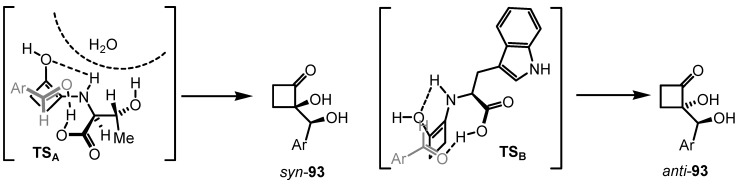
Rationalized transition states A and B for the L-Thr (TS_A_) and L-Trp (TS_B_) catalyzed aldol reaction of hydroxycyclobutanone **92** with aromatic aldehydes.

Enantioselective aldol reaction between 2-hydroxycyclobutanone **92** and aromatic aldehydes in DMF has been achieved using (*S*)-tryptophan **94** by Frongia and Ollivier. The reaction is completely regioselective and gives the 2,2-disubstituted cyclobutanone **93** in up to 80% yield [88]. The major adduct was obtained in 67% *ee*, with an *anti*-relative configuration in contrast with the selectivity of organocatalysed aldol reactions conducted on acyclic hydroxyketone substrates [89]. *anti*-Configuration was assigned by X-ray analysis and rationalized on the basis of an hydrogen-bonding interaction between the N–H and the cyclobutanone-alcohol function in the enamine intermediate (Figure 2 TS_A_). In this assumption, the approach of the aldehyde in the transition state is facilitated by the carboxylate function, which is preferentially oriented to minimize steric repulsion, leading to an *anti*-configuration of the aldol product *anti*-**93** (Scheme 24). 

Analogous reactions carried out in solvent-free conditions, using (*S*)-threonine, were investigated by the same group [90]. Deracemized aldol adducts featuring a chiral quaternary center were obtained in up to 72% yield, with *syn*-selectivity up to 85:15 dr and *ee* up to 84%. Switch on *anti/syn* configuration of compounds **93** were rationalized by the formation of a stabilizing hydrogen bonding network between the enamine-specie and the aldehyde acceptor as described in Figure 2 (TS_B_).

### 6.4. Organocatalyzed Mannich Addition of Cyclobutanones to Glycolates

**Scheme 25:**
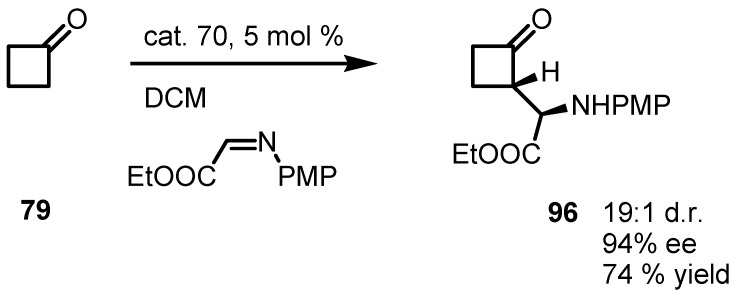
Enantioselective organocatalyzed Mannich reaction of cyclobutanone with glycolates.

Furthermore, (*S*)-pyrrolidinetetrazole **70** well catalyzed the Mannic reaction of cyclobutanone **79** with PMP-ethylglyoxylate imine affording the corresponding α-aminoacilcyclobutanone **96** in good yields [83,91,92] and *ee* as reported in Scheme 25.

**Scheme 26:**
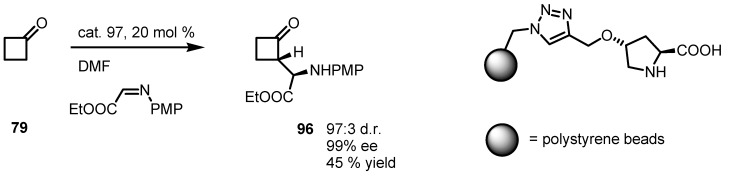
Enantioselective organocatalyzed Mannich reaction of cyclobutanone with glycolates.

Supported (*S*)-proline catalysts **97** were used by Rodriguez-Escrich and co-workers in the enantioselective Mannich reaction of different carbonyl compounds, including cyclobutanone **79** and *N*-PMP-ethylglyoxylate imine in the presence of the supported catalyst **97** [93]. In this investigation, the effect of the proline-support linker were studied; and 1,2,3-triazole linker constructed from azidomethyl polystyrene and *O*-propargyl hydroxyproline turned out to be optimal catalyst, both in terms of catalytic activity and enantioselectivity. With this protocol, compound **79**, was converted into the corresponding α-aminoacylcyclobutanone **96** in moderated yield, accompanied by high *ee* and excellent dr as reported in Scheme 26. 

### 6.5. Organocatalyzed Michael addition of Cyclobutanones to Nitrostyrenes

**Scheme 27:**
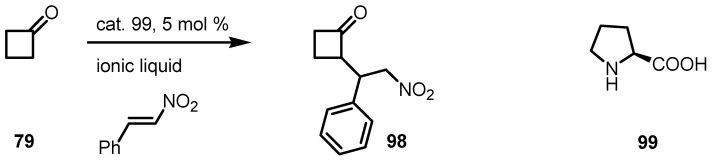
Organocatalyzed Michael reaction of cyclobutanone with nitrostyrenes.

Reaction of cyclobutanone **79** with nitrostyrene in presence of catalyst **99** and ionic liquids-[bmim]PF_6_ and [hmim]BF_4_ gave the racemic corresponding cyclobutanone-nitroadduct **98** in 43% yield [94] as described in Scheme 27. 

**Scheme 28:**
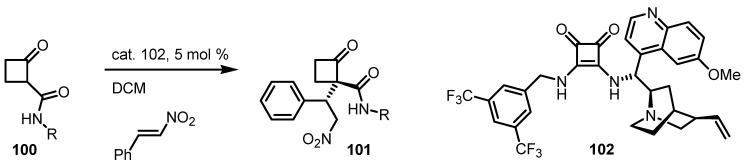
Enantioselective functionalization of 2-substituted cyclobutanones via Michael reaction.

Rodriguez and co-workers published in 2012 a straightforward, highly efficient diastereo- and enantioselective organocatalytic Michael additions of 2-substituted cyclobutanone derivatives **100** and nitroalkenes, affording the stereocontrolled creation of α-2,2-disubstituted cyclobutanone quaternary centers [95]. The approach relies on both the use of Brønsted base/hydrogen-bonding donor bifunctional organocatalysts **102**, based on cinchona alkaloids and importantly, the specific stabilization and activation of cyclobutanone with a secondary amide moiety. The reaction was found to nicely accommodate a broad scope of substrates, allowing the control of up to three contiguous stereogenic centers yielding the corresponding cyclobutanone derivatives **101** (Scheme 28) in excellent yields and *ee*. 

### 6.6. Cyclobutanone α-Heteroatom Functionalization

**Scheme 29:**
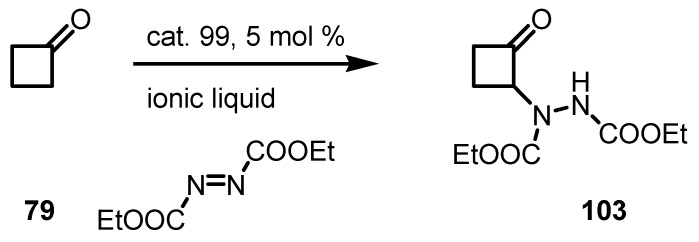
Heteroatom functionalization of cyclobutanone with azodicarboxylates.

Toma and co-workers reported the addition of cyclobutanone **79** to diethyl azodicarboxylate in ionic liquids-[bmim]PF_6_ and [hmim]BF_4_ in the presence of (*S*)-proline **99** [96]. The procedure afforded α-*N*-functionalized cyclobutanone **103** in moderate yields but no *ee* value was reported (Scheme 29). 

Better results were achieved when the same reaction was extended to different cyclic ketones or aldehydes. 

## 7. Cyclobutane Ring Enlargement

### 7.1. Chiral Non Racemic Cyclobutanes Ring Expansion

**Scheme 30:**
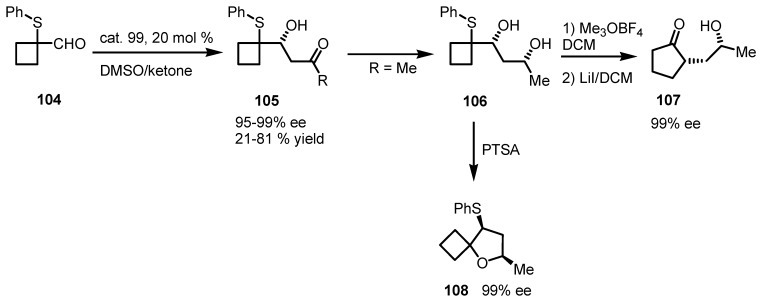
Enantiomerically enriched cyclobutane diols ring enlargement and spiranization.

Acid-catalyzed ring expansion of chiral cyclopropyl and cyclobutyl derivatives was reported from Piras and co-workers for the synthesis of strained carbo- and heterocyclic compounds [97]. Chiral adducts **105** were prepared using (*S*)-proline catalyzed direct asymmetric aldol reactions of 1-phenylthiocyclobutane carboxaldehydes **104** with different ketones. The aldol compounds were diastereoselectivelly reduced to diols **106** and transformed in the corresponding spirocyclic cyclobutane derivatives **108** using catalytic amounts of PTSA. Also, diol-adducts **106** were transformed into oxaspiroexanes by using Me_3_OBF_4_ which undergo ring expansion to chiral cyclopentanones **107** in high yields and *ee* values up to 99% as shown in Scheme 30. 

**Scheme 31:**
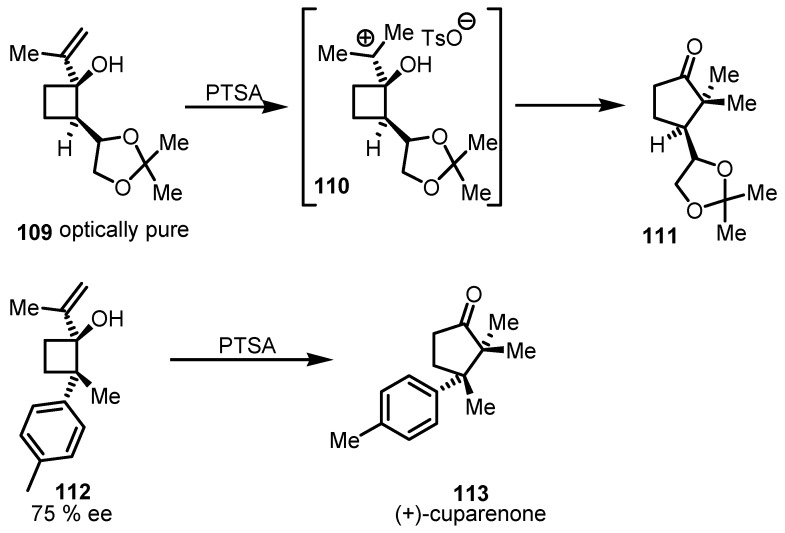
Acid catalyzed ring expansion of enantiomerically enriched cyclobutanols.

Geminal 2,2-dimethyl and 2,2-dialkylcyclopentanone **111** was prepared by acid catalyzed ring expansion of isopropenylcyclobutanol **109** by Piras and co-workers [98]. The stereochemical behaviour of this 1,2-sigmatropic shift clearly showed that, the pinacol-type rearrangement, occurred through carbocationic specie intermediate **110** without any detrimental effect on the optical purity of the starting allylic alcohols. The reaction was found to nicely accommodate a broad scope of substrates, allowing the control of the new stereogenic centers yielding the corresponding cyclopentanone derivatives (Scheme 31) in excellent yields and *ee*. The method was extended to differently substituted aryl cyclobutanols such as **112** that once submitted to acid-catalyzed ring expansion allowed to access to the family of sesquiterpene (+)-cuparenone (**113**, [99]) in good yields.

### 7.2. Chiral Non Racemic Oxaspirohexanes Ring Enlargement

Asymmetric epoxidation of benzylidenecyclobutane **114** and subsequent rearrangement, was performed by Shi and co-workers [100]. The synthetic protocol is based on a catalytic epoxydation of benzylidene derivatives **114** using fructose oxazolidinone **117** and oxone^®^ [101,102,103], affording the corresponding oxaspirohexanes **115** in high *ee* and good to excellent yields. 

**Scheme 32:**
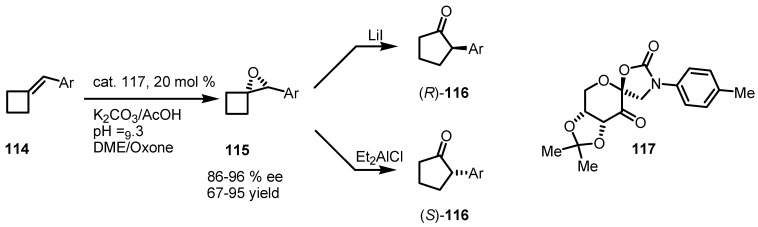
Enantioselective synthesis of 2-aryl cyclopentanones.

The so obtained chiral oxyranes were sucessfully transformed into the corresponding cyclo-pentanones (*R*)-**116** and (*S*)-**116** by using Lewis acids, such as Et_2_AlCl and LiI [104], achieving the enantiocontrolled ring expansion with the possibility to obtain the two enantiomerically enriched cyclopentanone enantiomers (Scheme 32).

### 7.3. Organocatalyzed Enantioselective Cyclobutane Ring Expansions

**Scheme 33:**
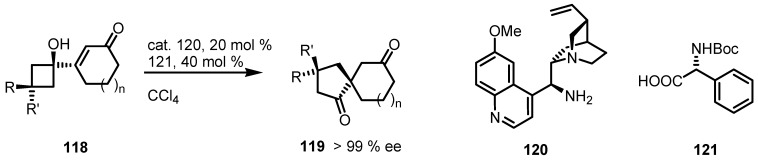
Enantioselective construction of chiral quaternary stereocentres in spirocyclic diketones.

The formation of quaternary stereogenic carbons results an actractive challenge in many stoichiometric and catalytic transformation. Tu and co-workers [105] have, very recently developed a straightforwardring expansion, based on a 1,2-sigmatropic semipinacolic rearrangement. This enantioselective transformation has been performed with vinylogous ketones **118** using a combination of *N*-Boc-L-phenylglycine **121** and a cinchona alkaloid as catalyst **120** (Scheme 33) leading to chiral spirocyclic dienones **119** in good to excellent yields and high enantioselectivity.

**Scheme 34:**
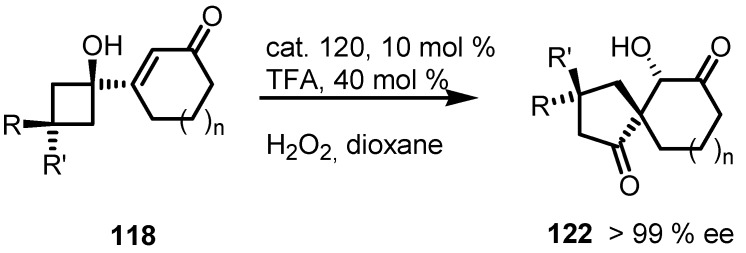
Synthesis of spirocycloalkanediones by organocatalytic asymmetric epoxidation.

Cinchona catalyst **120** has been used by the same group, in the synthesis of chiral spiroderivatives **122**, starting from cyclobutanols **118** [106]. This straightforward achievement has been obtained by using Brønsted acids (TFA) and hydrogen peroxide through a tandem enone epoxidation-cyclobutanol ring expansion, affording the spiroketoalcohols **122** in high yields and up to 99% *ee* (Scheme 34).

Tu and co-workers, also developed an interesting enantioselective organocatalyzed semipinacolic rearrangement of cyclobutanol allylic alcohols **123** [107]. This reaction represents a catalytic Paquette-type [108,109] cyclobutanol ring expansion, through the enantioselective protonation of dihydropyranyl- or furanyl double bonds, followed by a cyclobutanol sigmatropic 1,2-shift [110].

**Scheme 35:**
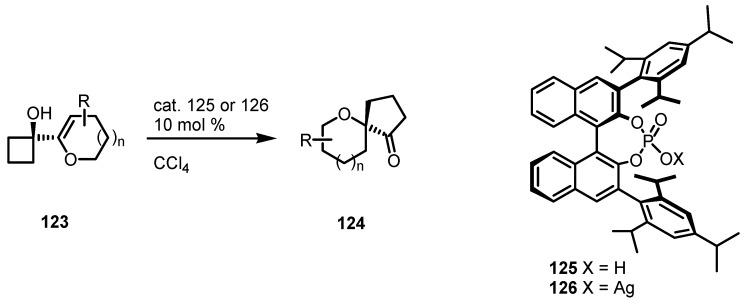
Enantioselective acid catalyzed ring expansion of cyclobutanols.

Bulky di-(2,4,6-triisopropylphenyl)-substituted phosphoric acid **125**, afforded the corresponding spirocompounds **124** with good enantioselectivity 74%–98% and good to excellent yields 51%–98%. Further modification of this protocol, involving silver phosphate **126** as catalyst, gave similar results as shown in Scheme 35. 

### 7.4. Organocatalyzed Enantioselective Fluorination-Induced Cyclobutanes and Cyclopropanes Ring Expansion

**Scheme 36:**
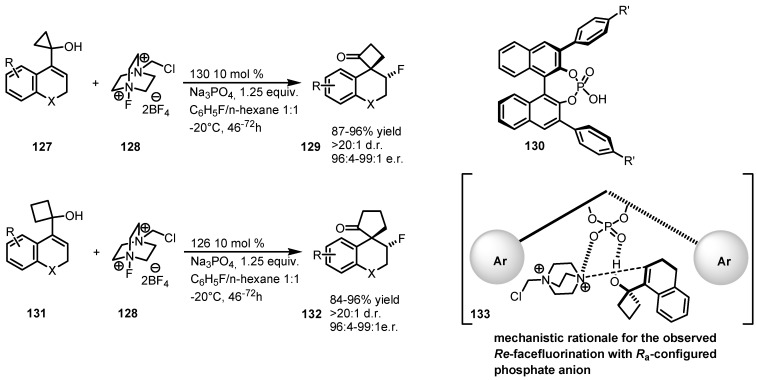
Enantioselective fluorination-induced cyclopropane and cyclobutane ring expansion.

Alexakis and co-workers developed an enantioselective organocatalyzed fluorination-induced Wagner-Meerwien rearrangement of allylic cyclopropanols 127 and cyclobutanols 131 [111]. This tandem fluorination-ring enlargement reaction was achieved by using binol-derived phosphoric acids 130 in the presence of the fluorinating agent 128. Phosphoric acids 130 were used as a privileged source of chiral anions, able to induce asymmetry through an interaction between the strained allylic alcohols 131 (or 127) and 128 as reported in the mechanistic rationale 133 (Scheme 36). β-Fluoro spirocyclic ketones 129 and 132 were isolated in high yields and *ee*, also accompained by excellent levels of diastereoselection. 

## 8. Enantioselective Bayer-Villiger Oxidation of Cyclobutanones

### 8.1. Enantio- and Diastereoselective Bayer-Villiger Oxidation of Chiral Cyclobutanones

**Scheme 37:**
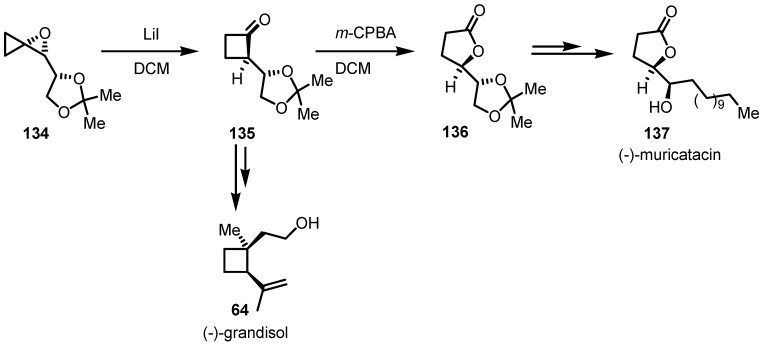
Enantio- and diastereoselective Bayer-Villiger oxidation of cyclobutanones.

Enantiomerically enriched oxaspiropentane **134** were transformed into the corresponding cyclobutanone derivatives **135** [6,7] using LiI [104]. This investigation, allowed for the first time to understand the LiI intermediacy in the semipinacolic C3-C4 ring expansion through a double inversion of configuration process leading to the formation of the corresponding chiral cyclobutanones in high yields and *ee*. This strategy, was subsequently used by the same group for the synthesis of the enantiomerically enriched (−)-grandisol (**64**, [112]) and (−)-muricatacin **(137**, [113]) pheromones as reported in Scheme 37.

### 8.2. Organocatalyzed Enantioselective Cyclobutanone Bayer-Villiger Oxidation

**Figure 3:**
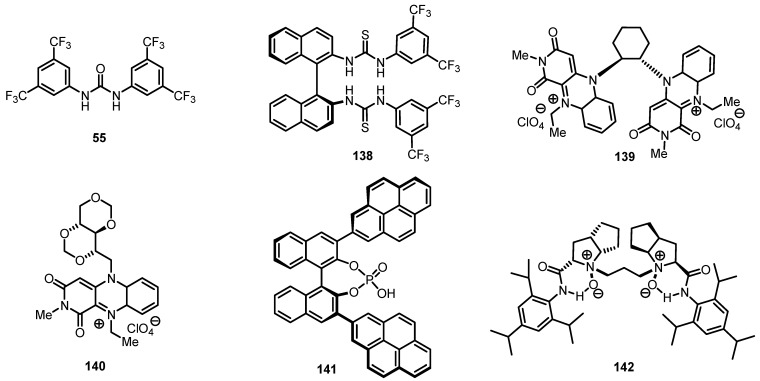
chemo- and enantioselective cyclobutanone Bayer-Villiger metal-free catalysts.

Enantioselective Bayer-Villiger oxidation has been performed by different groups in the last years and a certain number of chiral ligands and catalysts have been synthesized and explored (Figure 3). Cyclobutanone moiety, is relatively easy to oxidize, due to the ring strain of the carbocyclic system [1,2] and attempts to catalyze this oxidation reaction are well documented [114,115,116,117,118,119]. Thiourea based catalysts **55** and **138** [120], are able to catalyze efficiently the oxidation of 3-substituted cyclobutanones **143**, affording the corresponding lactone derivatives **144** in good yields. 

More recently, good results were achieved using chiral flavine derivatives **139** [121] and **140** [122] (Figure 2) in association with hydrogen peroxide, affording the corresponding lactones in good yields and *ee* (61%–74% *ee*). Better results were obtained using chiral phosphoric acids **141** and hydrogen peroxide isolating the corresponding lactones **143** in high yields and good *ee* (55%–93%) [123,124]. 

**Scheme 38:**
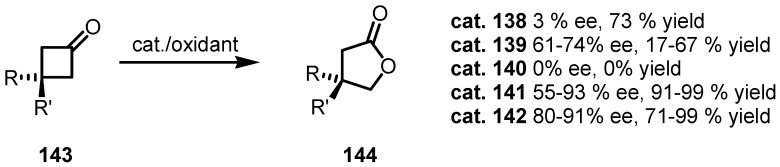
Organocatalyzed enantioselective cyclobutanone Bayer-Villiger oxidation.

Moreover, bis-pyrrolidineoxide ligand **142** was used in this reaction obtaining straightforward cyclobutanone oxidation to **144** in high yields and high *ee* (80%–91%) [125]. Results of these investigations are reported in Scheme 38. 

### 8.3. Biocatalytic Enantioselective Cyclobutanone Bayer-Villiger Oxidation

**Scheme 39:**
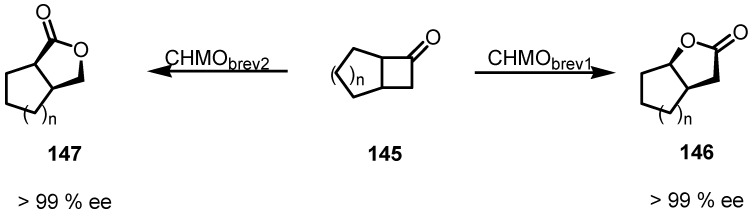
Biocatalytic enantioselective cyclobutanone Bayer-Villiger oxidation.

An interesting Microbial Baeyer–Villiger oxidations of fused bicyclic ketones **145** with a cyclobutanone structural motif has been reported. Enantioselective cyclobutanone oxidation catalyzed by recombinant Escherichia coli cells was performed using monooxygenases from Brevibacterium, CHMObrev1 and CHMObrev2 [126] leading to γ-lactones as reported in Scheme 39. 

Interestingly, the two CHMO forms allowed to achieve different regiochemical results, obtaining the conventional Bayer-Villiger lactone **146** through the migration of the more substituted carbon atom. However, once CHMObrev2 was used, lactones **147**, were isolated, resulting from the migration of the less substituted carbon atom. 

Phenylacetone Monooxygenase PAMO mutants were also screened as potential Bayer-Villiger oxidation biocatalysts in the transformation of cyclobutanone **148** in the corresponding lactone **149** [127]. 

**Scheme 40:**
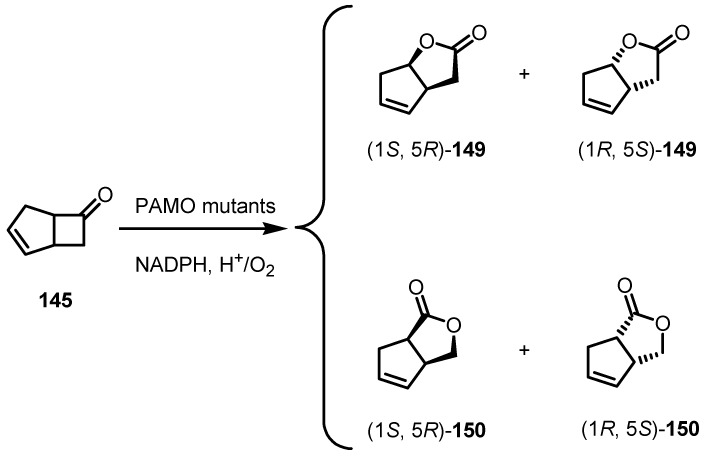
PAMO-mutants biocatalytic cyclobutanone Bayer-Villiger oxidation.

This unusually thermostable enzyme, is a promising candidate for catalyzing enantioselective Baeyer-Villiger reactions in organic chemistry. Unfortunately, however, its substrate scope is very limited, reasonable reaction rates being observed essentially only with phenylacetone and similar linear phenyl-substituted analogs. The oxidation of substrate **148** proceeds with the preferential formation of lactone (1*S*,5*R*)-**149**, whereas the mutants lead to a reversal of enantioselectivity (Scheme 40).

## 9. Conclusions

Cyclobutane and cyclobutanone are easily accessible and useful synthetic tool that still represent a challenging target for organic chemists. Enantioselective stoichiometric and catalytic approaches have been developed with successful results, giving to this class of compounds a relevant role as key intermediate in a large number of asymmetric synthetic applications. Based on these findings, it is reasonable to expect that new interesting methodologies focused on the use of this versatile family of compounds will soon be developed. 
